# Joint Angle Estimation of a Tendon-Driven Soft Wearable Robot through a Tension and Stroke Measurement

**DOI:** 10.3390/s20102852

**Published:** 2020-05-17

**Authors:** Byungchul Kim, Jiwon Ryu, Kyu-Jin Cho

**Affiliations:** 1 Biorobotics Laboratory, School of Mechanical and Aerospace Engineering, Seoul National University, Gwanak-ro 1, Gwanak-gu, Seoul 08826, Korea; kbc1990@snu.ac.kr (B.K.); jiwon.ryu@snu.ac.kr (J.R.); 2 Soft Robotics Research Center, Seoul National University, Gwanak-ro 1, Gwanak-gu, Seoul 08826, Korea

**Keywords:** soft wearable robot, robotic systems parameter estimation, joint angle estimation, data-driven control

## Abstract

The size of a device and its adaptability to human properties are important factors in developing a wearable device. In wearable robot research, therefore, soft materials and tendon transmissions have been utilized to make robots compact and adaptable to the human body. However, when used for wearable robots, these methods sometimes cause uncertainties that originate from elongation of the soft material or from undefined human properties. In this research, to consider these uncertainties, we propose a data-driven method that identifies both kinematic and stiffness parameters using tension and wire stroke of the actuators. Through kinematic identification, a method is proposed to find the exact joint position as a function of the joint angle. Through stiffness identification, the relationship between the actuation force and the joint angle is obtained using Gaussian Process Regression (GPR). As a result, by applying the proposed method to a specific robot, the research outlined in this paper verifies how the proposed method can be used in wearable robot applications. This work examines a novel wearable robot named Exo-Index, which assists a human’s index finger through the use of three actuators. The proposed identification methods enable control of the wearable robot to result in appropriate postures for grasping objects of different shapes and sizes.

## 1. Introduction

Due to significant improvements in actuation and sensing components in terms of size and performance, technologies for wearable devices (e.g., haptic devices [1], wearable sensors [2,3], wearable robots [4,5,6,7]) have received great attention and have been developed for various purposes. Development of these devices requires an in-depth understanding of human properties, because these devices are intended to be worn on the human body. For instance, in the case of wearable robots, differences in the size and shape of the bone structure of different potential human users should be considered in the robot design. Further, shape and stiffness of joints are also important in body motion that is assisted by a robot. Since these human factors vary from person to person, developing robots with consideration of these factors has been a difficult problem for researchers.

One way to address these issues is to use soft material; softness provides adaptability and is more comfortable to wear [8,9,10]. In this approach, the size effect can be easily handled because a soft structure can fit well against the human body, even if there is a slight difference in size. The use of soft material also makes soft robots more compact; this is because—due to the inherent characteristics of soft wearable robots—there are no joint alignment issues. Joint alignment issues in rigid robots are a safety concern; efforts to minimize these issues result in added size [11,12]. In order to sustain the advantages of these material properties, tendons are often used as the transmission in soft robots; using this approach, rigid and bulky parts such as a battery, actuators, and controllers can be located at a distance from the wearable part [13,14] and force can be easily applied on several joints with fewer actuators [15]. Therefore, numerous hand wearable robots have been developed using soft materials and tendon transmissions. Among body parts, the hand is one of the most effective potential areas for the use soft components and tendon transmissions; this is because the hand has many joints in a compact space.

For instance, In et al. developed a tendon-driven soft robotic glove, named SNU Exo-Glove, for people with spinal cord injuries [4]. This glove assists the thumb, index, and middle finger through the use of three actuators. For adaptability, an under-actuation mechanism is applied in this robot. Exo-Poly was developed by the same research team; in this work they changed the material of the robot body from garment to polymer [16]. Other researchers developed Graspy Glove for high portability, by attaching all actuators and electrical circuits on the back side of the hand [17]. For rehabilitation purposes, BiomHED was developed by mimicking the muscle and tendon system of the human hand [18]. This robot tries to mimic the natural postures of the human hand by attaching wires similar to the tendon of the human hand. A commercially developed glove, named Gloreha, has also been developed to provide rehabilitation through a virtual reality environment [19,20].

However, the adaptability of a robot generated with a soft structure and tendon transmission could cause difficulties in wearable robot control; this is because these approaches increase the uncertainties, which originate from both elongation of the soft material and from undefined human properties [21,22]. For example, soft structure elongates when force is applied; this elongation induces difficulty in estimating the acting point and the direction of the force. Also, estimation of the joint angle using actuation information is not easily solved. This is because most tendon-driven hand wearable robots actuate several joints via a smaller number of actuation wires for compactness. In these cases, numerous joints rotate even when only a single wire is pulled; therefore, the joint angle cannot be decided using only the wire stroke. Instead, the position of the joints are decided by joint stiffness, wire moment arm, and wire tension; these are not easy to estimate due to human uncertainties and uncertainties from the elongation of soft materials.

As an alternative to the estimation, the method of using wearable sensors can be considered. One example is attaching strain sensors on the surface of the body. The strain sensor measures the angle of rotation by measuring the change of electric resistance during elongation and compression. Park et al. [23] developed a strain sensor with a novel fabrication method known as inkjet-printed SWCNT (Single-walled Carbon Nanotube) film, and this strain sensor can bear the strain up to 80% while others can generally bear up to 5%. This method, however, has a significant weakness under change in humidity. Another wearable sensor which can be used to measuring the rotation angle at the finger joints is IMU sensors [1,24,25]. A typical problem of an IMU sensor is the accumulation of the error in position over time because it integrates the acceleration along the time. Another issue is the discomfort that the wearer feels due to the sensors attached. Moreover, IMU sensors are prone to get affected by external noises and magnetic fields generated by nearby objects. Using RGB-Depth cameras and motion capture systems can also be an alternative method for measuring the joint angles. Zhao et al. [26] constructed three-dimensional hand shape using multiple Vicon cameras and a Kinect camera. Some other works dealt with the problem using only a single RGB-D camera and then classified each segment in hand by neural networks [27,28]. However, methods relying solely on visual data still showed major accuracy problems under occlusions.

In this paper, we propose a soft robotic glove named Exo-Index, which is controlled with consideration of the uncertainties that arise from human factors and the robot’s soft components without any wearable sensors. In order to deal with these uncertainties, a data-driven method is used to identify both kinematic information and stiffness parameters of the system. For the kinematic identification, the exact joint position, the ground truth, is measured using vicon motion capture cameras. Here, unlike previous human kinematic studies [29], joint position is estimated as a function of joint angle to increase accuracy. The concept of Product of Exponential (POE) is used in this estimation as well [30]. In the stiffness parameter estimation, the relationship between wire tension, wire stroke and joint angle is obtained by measuring the wire tension, wire stroke and joint angle simultaneously. With the synchronized data, estimation is proceeded using Gaussian Process Regression (GPR) [31]. Using this method, this paper also shows that Exo-Index can make different grasps, to effectively grasp different object shapes. The robot supports three major grasps, specifically wide pinch, narrow pinch, and caging.

The remainder of this paper is organized as follows. First, details of the proposed robot design and the method to identify both kinematic and stiffness parameters of the system are described in Section 2. The results of kinematic identification and stiffness parameter estimation, along with robot performance using the proposed methods, are explained in Section 3. A discussion of the research is found in Section 4. Finally, the conclusion is explained in Section 5. In addition to the main text, additional information is provided in the Appendix A; Appendix A briefly explains human hand anatomy, which provides important background for the work described in this paper.

## 2. Materials and Methods

### 2.1. System Design

As explained in the Introduction section, Exo-Index is designed to assist a user in making three grasp types (i.e., wide pinch, narrow pinch, and caging) by controlling the tension of three wires. This section describes the detailed design methodology of the robot. Section 2.1.1 explains the design methodology of the proposed glove. Next, details of the controller and actuator design are described in Section 2.1.2.

#### 2.1.1. Glove Design

**Figure 1:**
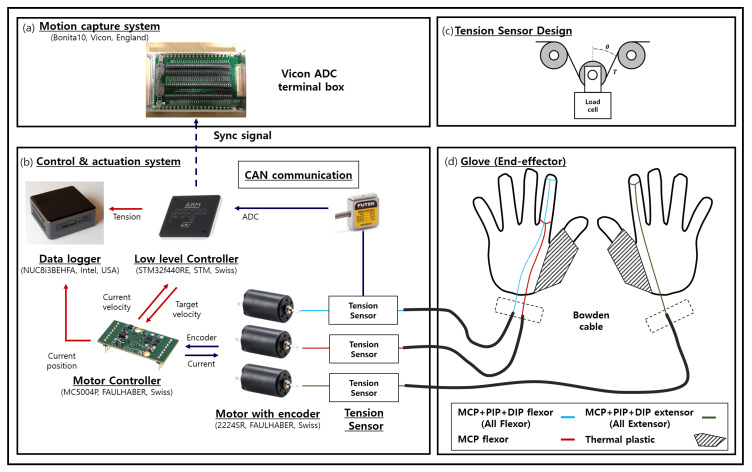
Schematic view of the Exo-Index system; (**a**) shows an external device for synchronizing load cell data measured in the MCU and posture data measured by the Vicon; (**b**) shows overall control and actuation system for the robot; (**c**) shows how the tension sensor is designed using loadcell; (**d**) provides a brief look at how the three tendons of the actuator are connected to the glove.

**Figure 2:**
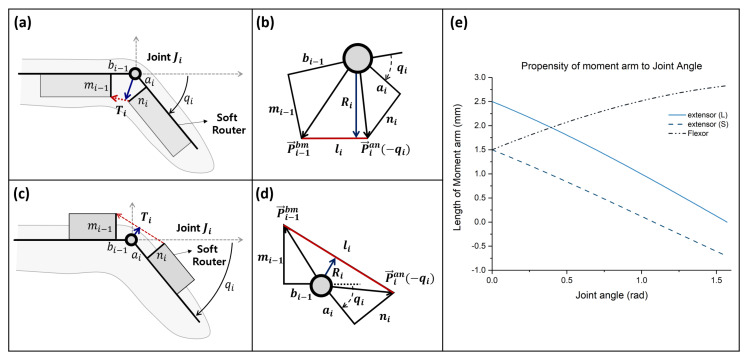
Schematic view of the tendon routing method in the Exo-Index; (**a**) and (**b**) show a schematic of the flexor router, while (**c**) and (**d**) show a schematic of the extensor router; (**e**) shows aspects of how the moment arm changes with respect to variation of the joint angle. Here, length of the flexor router and extensor (S) router ($a_{i}$, $b_{i-1}$, $m_{i-1}$, and $n_{i}$) each are 5, 5, 3, and 3 mm, while $m_{i-1}$, and $n_{i}$ of the extensor (L) router is increased to 5 mm.

In order for Exo-Index to assist with three types of grasp, two actuation wires (wires named MCP-flexor and Whole-flexor) are connected on the palmar side, and a single actuation wire (the wire named Whole-extensor) is connected on the dorsal side of hand, as shown in Figure 1. Here, the thumb is fixed in an opposed posture by using thermal plastic, as in previous research [16]. In the glove design process, it is important to determine the tendon path, because the path is related to the torque applied on the finger. Therefore, this section describes the analytical solution of the relationship between the torque applied on the joint and the wire tension. Although it is true that the accuracy of the analytical solution cannot be guaranteed due to the friction of the wire and the deformation of the garment, as explained in the introduction section, the analytical solution was sought because it provides insight into a method to determine the tendon path. In order to fix the wire path, it is possible to use bearings or conduits [15]. When bearings are used, torque applied on the joints is sustained equally because the moment arm and path of the wire does not change even as the joints move. On the other hand, if conduits are used to fix the wire path, the moment arm and path vary as the joint angle changes. Varying the moment arm can be problematic in a robotic system because it makes control difficult; however, the Exo-Index in this study used a conduit-type transmission because it has advantages in making the system compact, which is highly important in wearable robot applications. A schematic view of how the wire is fixed in the proposed robot system is depicted in Figure 2.

As shown in Figure 2, the path and moment arm of the tendon changes according to the joint angle. This is because routers rotate as the finger moves. The position of the router when the joint angle is $-q_{i}$ is simply derived using the rotation matrix, as shown in Equation (1), by using the initial position of the router shown in Figure 2.

(1) $${\vec{P}}_{i}^{\;an}\left(-q_{i}\right)=Rot\left(-q_{i}\right){\vec{P}}_{i}^{\;an}\left(0\right)=\left[\begin{array}{cc} \cos(-q_{i}) & -\sin\left(-q_{i}\right)\\ \sin\left(-q_{i}\right) & \cos(-q_{i}) \end{array}\right]{\vec{P}}_{i}^{\;an}\left(0\right).$$

Using the position of the soft router, the length of the moment arm can be derived by using the concept of the cross product, as in Equation (2).

(2) $$R_{i}=0.5\frac{norm({\vec{P}}_{i-1}^{\;bm}\times {\vec{P}}_{i}^{\;an}\left(-q_{i}\right))}{|{\vec{P}}_{i-1}^{\;bm}-{\vec{P}}_{i}^{\;an}\left(-q_{i}\right)|\;}.$$

Since the relationship between the joint angle and moment arm of the wire is non-linear, the finger configuration in terms of tension can be solved numerically, rather than analytically. One thing we can intuitively know about the relationship in Equation (2) is that the moment arm of the extensor wire could be shorter than that of the flexor, even if the router configuration is the same. For example, when the lengths of $a_{i}$, $b_{i-1}$, $m_{i-1}$, and $n_{i}$ are 5, 5, 3, and 3 mm, respectively, the moment arm of the flexor and extensor wire can be described as shown in Figure 2. As shown in the graph, the moment arm of the flexor increases as the joint angle increases, while that of the extensor reduces. Further, the moment arm of the extensor becomes negative when the angle increases; this means that even if the tension of the extensor increases, extension may not occur. In a real-world situation, thanks to the finger structure, the moment arm of the extensor can be sustained larger than zero because the finger skin will prevent the situation where the moment arm would become negative. However, this situation is quite unstable because sometimes the glove can deform and the wire path may rotate to the side direction; this causes the moment arm to be negative. Therefore, it is safe to make the extensor moment arm larger than zero. This is possible by increasing the height of the soft router ($m_{i-1}$ and $n_{i}$). For instance, when $m_{i-1}$ and $n_{i}$ of the router increase to 5 mm, it is possible to sustain the moment arm of the extensor larger than zero, even as the joint angle increases. Using these results, the wire path of the proposed robot is designed.

**Figure 3:**
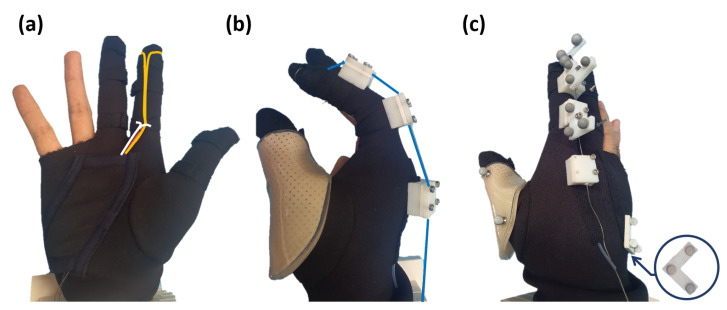
Overall view of Exo-Index: (**a**) shows two flexors (yellow line means All Flexor (A.F.) while the white line shows MCP Flexor (M.F.)) in a hand-open position, (**b**) shows an extensor named as All Extensor (blue line) in a hand-held position. In addition, the method of attaching vicon markers is shown in (**c**). Three markers attached on the back side of the hand are considered as the base frame.

Using the tendency between the moment arm and the joint angle in Figure 2, Exo-Index was developed, as shown in Figure 3a. In order to create a sufficient grasping force with less tension, the flexor was designed to pass through the bottom of the finger, thereby maximizing the moment arm of the flexor. Here, the path of the flexors was fixed by sewing the soft garment around the finger. For the extensor router design, a rigid component was used. Since moment arm of the extensor reduces as the joint angle increases, the path of the extensor was determined so that the moment arm is larger than zero even the finger is fully flexed, as shown in Figure 3b. Also, use of a rigid component as an extensor router not only serves the role of fixing the wire path, it also enables vicon markers to be fixed reliably, as shown in Figure 3c.

#### 2.1.2. Actuation and Control System Design

**Figure 4:**
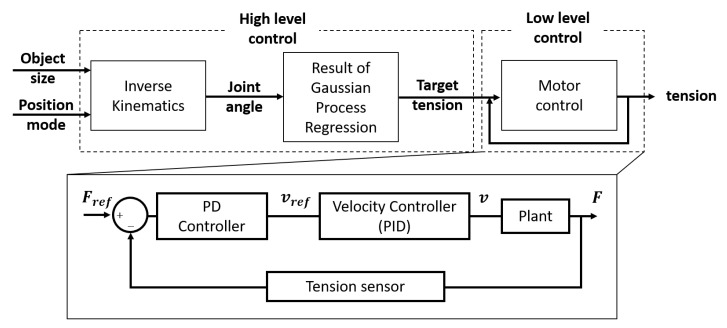
Block diagram of the control scheme used in the proposed robot.

The actuation system consists of three independent tendon driven actuators (FAULHABER, Croglio, Switzerland). These three actuators are controlled by a microcontroller unit (ST Microelectronics, Geneva, Switzerland) and three motor drivers (FAULHABER, Croglio, Switzerland) under CANopen communication. Overall control scheme of the proposed robot can be expressed as Figure 4. The high level control roles to find out the appropriate tension that makes grasp posture with given grasp mode. Since the proposed robot does not contain any additional vision sensor, the size of the object and grasp mode (e.g., narrow pinch, wide pinch, and caging) are decided manually. With given object size and grasp mode, target tension of three different actuators are induced by using the result of inverse kinematics and regression. After that, the tension is controlled by a low level controller using additional tension sensors designed with loadcells (FUTEK, Irvine, California, USA); Detail schematic of tension sensor with loadcell is depicted in Figure 1c [32] and θ in the schematic is 0 (rad) in our case. In this controller, admittance control is used; the tension is controlled by velocity, which is based on a PD controller with motor encoder, as shown in Equation (3) and Figure 4 [33].

The resolution and maximum non-linearity of the tension sensing unit can be described as 0.004 N and 0.2 N, respectively. It can be derived from the resolution (0.002 N) and maximum non-linearity (0.1 N) of the loadcell installed in the tension sensing unit. This is because the friction of the wire at the tension sensing unit is negligible and the tension sensing unit is designed to measure twice of the wire tension. In addition, the resolution of the motor encoder can be described as 16 lines per revolution. Since the motor has 69:1 gear ratio, we can conclude that the resolution of the motor encoder is about 0.006 rad.

(3) $$v_{ref}=k_{p}\left(F_{ref}-F\right)+k_{d}\dot{F}.$$

**Figure 5:**
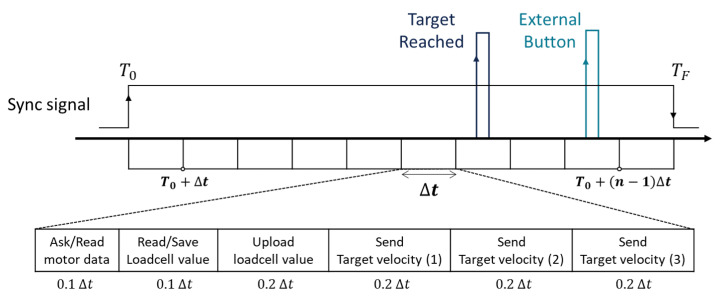
Schematic of time scheduling in the controller for reliable data acquisition.

Since the robot system must control three motors while syncing these data with vicon data, time scheduling in the control loop is important. When the system enters into a control state ($T_{o}$ in Figure 5), a sync signal is generated from the STM board and is transmitted to the vicon ADC terminal box. Next, the three motors are controlled every 20 ms (Δt). In order to avoid a situation in which the CAN Bus is overloaded, the time-interval (Δt) is then divided into 10 sub time-intervals (i.e., the STM chip is controlled at a frequency of 500 Hz.) In every sub time-interval, each task is then scheduled as shown in Figure 5. Since all data is transmitted through a single CAN Bus, it is possible to trust that the synchronization of the data has been done properly. Using the synchronized data, kinematic and stiffness parameter estimation proceeds, as explained in the next section.

### 2.2. Kinematic and Stiffness Parameter Estimation

**Figure 6:**
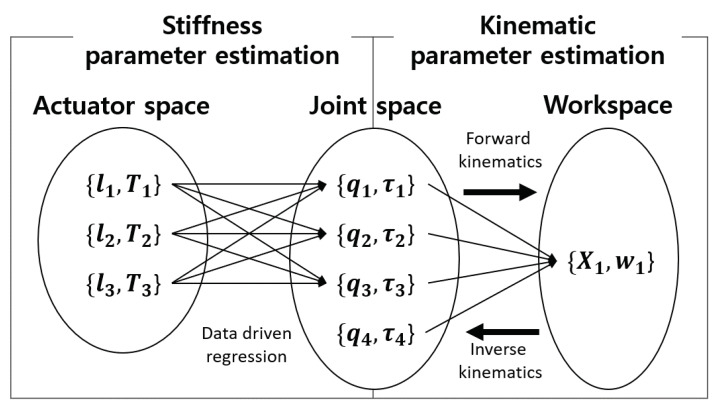
Schematic diagram to show purpose of kinematic identification and stiffness parameters estimation. Stiffness parameter estimation elucidates the relationship between the joint angle and the actuation data. The main purpose of stiffness parameter estimation is to obtain the relationship between the joint and the end-effector; this is highly related to kinematic analysis.

In order to make a target motion using Exo-Index, both kinematic information (finger length and joint position) and stiffness parameters (wire tension, finger joint stiffness, wire length) of the robot-human system must be identified, as shown in Figure 6. When the kinematic information is identified, it is possible to solve the relationship between the joint space and the work space using forward and inverse kinematics. In robot control, this enables a controller to calculate the target joint when the required fingertip position is given. Further, when the stiffness parameters are estimated, we can understand the relationship between the actuator space and the joint space. Using this relationship, the control system can calculate the target actuation force in terms of the target joint angle. Details about kinematic system identification and stiffness parameters estimation are explained in the following subsections.

#### 2.2.1. Kinematic System Identification: The Relationship between the Joint Angle and the Fingertip Position

In robotics, a relation between a joint angle and an end-effector can often be expressed through forward and inverse kinematics. In this subsection, we introduce a method to solve the kinematics of an index finger. We propose a method of finding the exact center of rotation in a finger joint using the position of vicon markers attached on the skin. In order to find the center of rotation, the product of exponentials method (POE) is used in this research.

(4) $$\left[\begin{array}{c} X_{M}\\ 1 \end{array}\right]=T_{MF}\left[\begin{array}{c} X_{F}\\ 1 \end{array}\right]=\left[\begin{array}{cc} R_{MF} & P_{MF}\\ 0 & 1 \end{array}\right]\left[\begin{array}{c} X_{F}\\ 1 \end{array}\right].$$

**Figure 7:**
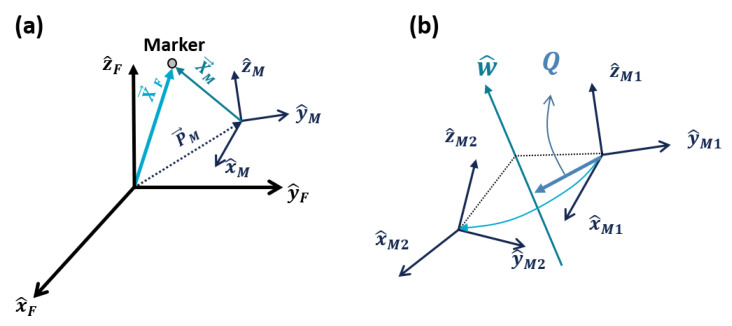
Schematic view to explain the transformation and rotation of coordinates.

First, the position of each marker measured directly from the Vicon expressed in a fixed frame **{F}** should be transformed with respect to a moving frame **{M}**, which moves along with the hand, since it is the relative position of the finger with respect to the hand that is meaningful. We defined this reference moving frame **{M}** to be located at the back of the hand (base frame in Figure 3), since this part does not move relative to other parts in a hand while an index finger is in motion. The coordinate systems of the fixed frame **{F}** and reference frame **{M}** are shown in Figure 7a. Using this concept, transformation of a marker position $X_{M}\in R^{3\times 1}$ from frame **{F}** into frame **{M}** can be written as Equation (4), using a transformation matrix $T_{\mathrm{MF}}$$\in R^{4\times 4}$. $X_{M}$, $X_{F}$, and $P_{M}$ are illustrated in Figure 7a, and $R_{\mathrm{MF}}$ is a rotation matrix from frame **{M}** to **{F}**.

(5) $$\begin{array}{cc} {\hat{x}}_{F} & =v_{xx}{\hat{x}}_{M}+v_{xy}{\hat{y}}_{M}+v_{xz}{\hat{z}}_{M}\\ {\hat{y}}_{F} & =v_{yx}{\hat{x}}_{M}+v_{yy}{\hat{y}}_{M}+v_{yz}{\hat{z}}_{M}\\ {\hat{z}}_{F} & =v_{zx}{\hat{x}}_{M}+v_{zy}{\hat{y}}_{M}+v_{zz}{\hat{z}}_{M} \end{array}$$

The basic concept of deriving the transformation matrix in Equation (4) is expressing the $X_{M}$ with respect to the frame **{F}**, as shown in Equation (6), while ${\hat{x}}_{F}$, ${\hat{y}}_{F}$, ${\hat{z}}_{F}$ can be written as Equation (5). From the relationship between the two coordinates shown in Equation (5), the position of the markers in frame **{M}** can be expressed in a matrix form, as shown in Equation (6). Other details about coordinate transformation can be found in previous works about robotics [30]. One thing different from traditional robotics is that usually a transformation matrix shows different forms because the matrices are defined to transform a vector from a moving frame to a fixed frame.

(6) $$\begin{array}{cc} {\vec{X}}_{M} & =X_{Mx}{\hat{x}}_{M}+X_{My}{\hat{y}}_{M}+X_{Mz}{\hat{z}}_{M}\\ \; & ={\vec{X}}_{F}-{\vec{P}}_{M}\\ \; & =X_{Fx}{\hat{x}}_{F}+X_{Fy}{\hat{y}}_{F}+X_{Fz}{\hat{z}}_{F}-\left(Pm_{x}{\hat{x}}_{F}+Pm_{x}{\hat{y}}_{F}+Pm_{x}{\hat{z}}_{F}\right)\\ \; & =\left(X_{Fx}-Pm_{x}\right)\left(v_{xx}{\hat{x}}_{M}+v_{xy}{\hat{y}}_{M}+v_{xz}{\hat{z}}_{M}\right)\\ \; & +\left(Y_{Fy}-Pm_{y}\right)\left(v_{yx}{\hat{x}}_{M}+v_{yy}{\hat{y}}_{M}+v_{yz}{\hat{z}}_{M}\right)\\ \; & +\left(Z_{Fz}-Pm_{z}\right)\left(v_{zx}{\hat{x}}_{M}+v_{zy}{\hat{y}}_{M}+v_{zz}{\hat{z}}_{M}\right)\\ \; & =\left[\begin{array}{ccc} v_{xx} & v_{yx} & v_{zx}\\ v_{xy} & v_{yy} & v_{zy}\\ v_{xz} & v_{yz} & v_{zz} \end{array}\right]\left(X_{F}-Pm\right)\\ \; & =R_{MF}\left(X_{F}-Pm\right)\\ \; & =R_{MF}X_{F}+P_{MF}. \end{array}$$

A transformation matrix can also be expressed as products of exponential (POE) by introducing a screw axis $S\in R^{6\times 1}$. A screw axis S is equal to ${\left[w,v\right]}^{T}$, where w and v refer to an angular and a linear velocity of a moving frame, respectively. Equation (7) is an example of a POE expression that can be used in the case illustrated in Figure 7b. Figure 7b shows a rotation of a moving frame $\left\{M_{1}\right\}$ to the frame $\left\{M_{2}\right\}$ about a rotational axis $\hat{w}$. In Equation (7), $M_{M_{1}M_{2}}$ refers to the transformation matrix from frame $\left\{M_{1}\right\}$ to $\left\{M_{2}\right\}$ at their initial position, and $e^{[s]\theta }$ in Equation (8) refers to the transformation that actually occurs due to the rotation, while $[S]=\left[\begin{array}{cc} \left[w\right] & v\\ 0 & 0 \end{array}\right]\in R^{4\times 4}$ is a matrix form of S, and θ is an angle of rotation. Q directs to an arbitrary point on the rotational axis and is expressed in frame $\left\{M_{1}\right\}$. In this sense, we can express the rotation from a coordinate to another along the screw axis.

(7) $$T_{M_{2}M_{1}}=e^{[S]\theta }M_{M_{2}M_{1}}$$

(8) $$e^{[S]\theta }=\left[\begin{array}{cc} e^{[\hat{w}]\theta } & G(\theta )v\\ 0 & 1 \end{array}\right]=\left[\begin{array}{cc} R & P\\ 0 & 1 \end{array}\right]$$

(9) $$\begin{array}{c} G\left(\theta \right)=I\theta +\left(1-\cos\theta \right)\left[\hat{w}\right]+\left(\theta -\sin\theta \right){\left[\hat{w}\right]}^{2}\\ G^{-1}\left(\theta \right)=\frac{1}{\theta }I-\frac{1}{2}\left[\hat{w}\right]+\left(\frac{1}{\theta }-\frac{1}{\theta }\cot\frac{\theta }{2}\right){\left[\hat{w}\right]}^{2}. \end{array}$$

Based on the relationship between a transformation matrix **T** using Vicon data and matrix **T** using the POE method in Equation (7), we can finally estimate the center of rotation in a finger joint as a function of the rotation angle. The angle of rotation θ and rotational axis $\hat{w}$ can be obtained from Equation (10) and Equation (11). Here, $r_{ii}$ in Equation (10) is an *i*th component in the main diagonal of a rotation matrix and $w_{x},w_{y},w_{z}$ in Equation (11) are x, y, and z components of $\hat{w}$. From Equation (8), the linear velocity is equal to $G^{-1}\left(\theta \right)p$, while $G^{-1}\left(\theta \right)$ can be written as Equation (9). Knowing that the linear velocity is equal to −w×Q, we can determine the direction and magnitude of the Q, which points at the joint center, as in Equation (12) and Equation (13). Equation (12) and Equation (13) are based on an assumption that we are looking for the vector Q that is perpendicular to the rotational axis $\hat{w}$. This method can be applied to finding the center of the finger joints.

(10) $$\theta =\cos^{-1}\left(\frac{r_{11}+r_{22}+r_{33}-1}{2}\right)$$

(11) $$\left[\hat{w}\right]=\left[\begin{array}{ccc} 0 & -w_{z} & w_{y}\\ w_{z} & 0 & -w_{x}\\ -w_{y} & w_{x} & 0 \end{array}\right]=\frac{1}{2\sin\theta }\left(R-R^{T}\right)$$

(12) $$\hat{Q}=\hat{w}\times \hat{v}$$

(13) $$\left\|Q\right\|=\frac{\left\|v\right\|}{\|\hat{w}\|}.$$

In the case of an MCP joint, which has two degrees of freedom, instead of using Equation (7) we should use Equation (14) when expressing the transformation matrix. Equation (14) consists of products of exponentials based on two rotational axes, each of which are abduction and flexion, respectively. Since the transformation matrix measured from the Vicon data includes both abduction and flexion information, we should separate it into two different rotations and find out the rotation angles for each.

(14) $$T_{MCP}=e^{\left[S_{1}\right]\theta_{1}}e^{\left[S_{2}\right]\theta_{2}}M_{initial}.$$

Here, we introduce a numerical method to estimate $\theta_{1}$ and $\theta_{2}$ using the space Jacobian $J_{s}\left(\theta \right)$. This method starts by setting the initial guesses of $\theta_{1}$ and $\theta_{2}$ as $\theta_{initial}\in R^{2\times 1}$. Then we define a matrix $[A]\in R^{4\times 4}$ as Equation (15). $T(\theta_{\mathrm{initial}})$ is a transformation matrix with $\theta_{initial}$ as an input, and $T^{-1}$ is calculated from the ground truth data measured by Vicon. The next step is to calculate Δθ from Equation (16). The space Jacobian $J_{s}\left(\theta \right)$ can be calculated as shown in Equation (17) and Equation (18). Finally, we can update $\theta_{initial}$ by $\theta_{initial}+\Delta \theta$ and repeat the whole process until θ converges. In this way, we can determine the abduction and flexion angle separately at the MCP joint, and hence, we can also calculate the distance between two adjacent joints, which can also be regarded as the length of phalanges. More detailed information about the numerical method used in this process is elaborated in [30].

(15) $$\left[A\right]=log(T\left(\theta_{initial}\right)T^{-1})$$

(16) $$-A=J_{s}\left(\theta \right)\Delta \theta$$

(17) $$J_{s}\left(\theta \right)=\left[S_{1}|{S_{2}}^{\prime }\right]$$

(18) $$\begin{array}{cc} {S_{2}}^{\prime } & =Ad_{e^{[S1]\theta 1}}\left(S_{2}\right)\\ (\mathrm{where},\;Ad_{T}\left(S\right) & =\left[\begin{array}{cc} R & 0\\ R & R \end{array}\right]S\in R^{6\times 6}). \end{array}$$

#### 2.2.2. Stiffness Parameter Estimation: Relationship between Tension and Joint Angle

In order to calculate the relationship between the joint angle and wire tension, the moment arm obtained in Equation (2) can be used to calculate torque applied on the joint, as shown in Equation (19), where κ means joint stiffness and *I* means inertia of the finger. In most finger modeling, the term $I\ddot{q_{i}}$ in the equation is usually ignored because both *I* and $\ddot{q_{i}}$ are small (i.e, the force equation of the finger can be calculated under a quasi-static condition.)

(19) $$\tau_{i}=R_{i}T-\kappa \Delta q_{i}=I\ddot{q_{i}}.$$

When the quasi-static condition is used, the joint angle of the finger can be simply expressed as Equation (20). However, since the joint stiffness(κ) is a human property that changes according to various factors (e.g., joint angle, age, sex, posture), solving Equation (20) is not a simple problem.

(20) $$\Delta q_{i}=\frac{R_{i}T}{\kappa }.$$

Extending Equation (20) to the configuration of an entire finger, we can obtain the relationship between tension and the joint angle, as shown in Equation (21). In this equation, values in column 3 of matrix J are all negative because these are related to the extension wire, which applies opposite directional torque. $T_{A.F},T_{M.F},and\;T_{A.E}$ in the equation are the tensions of the All Flexor, MCP Flexor, and All Extensor wires, respectively. The meaning of All Flexor, MCP Flexor, and All Extensor can be found in Figure 1.

(21) $$\left[\begin{array}{c} q_{MCP}\\ q_{PIP}\\ q_{DIP} \end{array}\right]=J_{q,T}T=\left[\begin{array}{ccc} \frac{R_{11}}{\kappa_{1}} & \frac{R_{12}}{\kappa_{1}} & -\frac{R_{13}}{\kappa_{1}}\\ \frac{R_{21}}{\kappa_{1}} & 0 & -\frac{R_{23}}{\kappa_{2}}\\ \frac{R_{31}}{\kappa_{1}} & 0 & -\frac{R_{33}}{\kappa_{3}} \end{array}\right]\left[\begin{array}{c} T_{A.F}\\ T_{M.F}\\ T_{A.E}. \end{array}\right]$$

Since the $R_{ij}$ and $K_{i}$ in matrix $J_{q,T}$ are not constant, but rather are a function of *q*, Equation (20) cannot be solved with a method using an inverse matrix. However, an inverse matrix of $J_{q,T}$ is obtained as shown in Equation (22) based on an assumption that $R_{ij}$ and $K_{i}$ are just constants; this is for the purpose of finding the tendency, even if it is not accurate. Using the inverse matrix of $J_{q,T}$, we can see several finding—(1) If we want a posture where only $q_{DIP}$ is not zero, while $q_{MCP}$ and $q_{PIP}$ is zero, several design constraints are required—$R_{21}R_{33}-R_{21}R_{31}$ and $R_{13}R_{21}-R_{11}R_{23}$ should be negative so as to not make the required tension negative, which is impossible in a tendon transmission. However, when $R_{21}R_{33}-R_{21}R_{31}$ becomes smaller than zero, the second column of $J_{q,T}^{-1}$ becomes negative. This means that it is impossible to make a posture that only bends the PIP joint when the device is developed to make the posture that only bends the DIP joint. (2) If we want to make a posture that only bends PIP joints, it is required to make $R_{21}R_{33}-R_{23}R_{31}$ and $R_{13}R_{31}-R_{11}R_{33}$ positive. Using these two statements, we can conclude that it is difficult to make both postures, a posture that only bends the DIP joint and a posture that only bends the PIP joint; therefore, we need to select one posture among these two postures. In the process of developing Exo-Index, we chose a posture that bends the PIP joint. This is because the posture that only bends the DIP joint can be used for grasping a large object; however, this could burden the user‘s hand because the device only assists the index finger. In addition, the human hand cannot make a posture that only bends the DIP joint. (3) For a situation where only the MCP joint is bent, this posture is possible when the $T_{M.F}$ is non-zero while sustaining the other components as zero. With these findings, we can infer that this tendon path is suitable for the Exo-Index, which aims to make wide pinch, narrow pinch, and caging postures by changing the tension distribution. For wide pinch, which only bends the PIP joint, it is possible to establish this by co-contraction of A.F. (All Flexor in Figure 1), M.F. (MCP Flexor in Figure 1), and A.E. (All Extensor in Figure 1) or, in some cases, co-contraction of A.F. and A.E. Also, by contracting M.F, the narrow pinch posture can be made while the contraction of A.F. makes the caging posture.

(22) $$\left[\begin{array}{c} T_{A.F}\\ T_{M.F}\\ T_{A.E} \end{array}\right]=\frac{1}{R_{21}R_{33}-R_{23}R_{31}}\left[\begin{array}{ccc} 0 & \kappa_{2}R_{33} & -\kappa_{3}R_{23}\\ \frac{\kappa_{1}\left(R_{21}R_{33}-R_{23}R_{31}\right)}{R_{12}} & \frac{\kappa_{2}\left(R_{13}R_{31}-R_{11}R_{33}\right)}{R_{12}} & \frac{\kappa_{3}\left(R_{11}R_{23}-R_{13}R_{21}\right)}{R_{12}}\\ 0 & \kappa_{2}R_{31} & -\kappa_{3}R_{21} \end{array}\right]\left[\begin{array}{c} q_{MCP}\\ q_{PIP}\\ q_{DIP}. \end{array}\right]$$

However, since matrix $J_{q,T}$ is not a constant, the analysis using the inverse matrix is not accurate. Therefore, a method using data driven regression was adopted for stiffness parameter estimation. The result of using stiffness parameter estimation can be found in the results section.

### 2.3. Experimental Methodology

**Figure 8:**
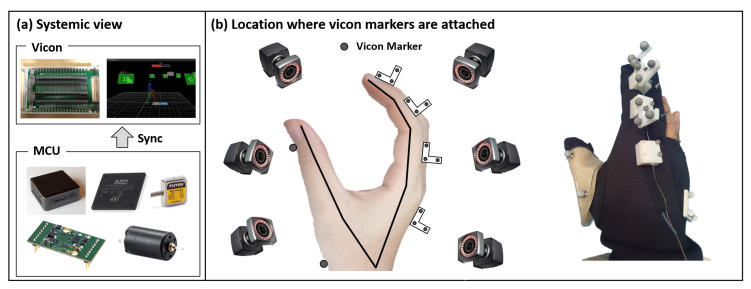
Schematic view of the experimental protocol: (**a**) shows a systemic view of vicon and the control system. By transmitting a sync signal to the ADC converter of the Vicon system, the loadcell data and encoder data were synchronized with the Vicon data. (**b**) shows the location where vicon markers are attached. A Total 14 markers are used in this experimental setup; 12 markers are used for the index finger and two markers are attached to the thumb to measure the thumb position.

The experiment was conducted for a single person as a pilot study because the main goal of this paper is to show how the robot was developed and controlled, rather than its clinical contribution. Here, the experiment was divided into two steps. The first experiment was conducted to find out the hand kinematics and the second experiment was designed for stiffness parameters estimation. In the first experiment, a total of 14 markers were used for hand motion tracking, as shown in Figure 8. Here, 12 markers were used to measure the position and orientation of the index finger, while the remaining two markers were used to measure the position of the thumb; Since we have to measure the MCP, PIP, DIP joint angle, three markers were attached to each phalange of the index finger as shown in the Figure 8. To measure the hand motion, eight motion capture cameras (Vicon, Hauppauge, Newyork, USA) were used. With this marker configuration, joint configuration was derived using the concept of forward and inverse kinematics. In the first experiment, the participant was asked to move all possible ranges when moving his finger spontaneously. After solving the kinematics of the hand, a second experiment was conducted to find out the relationship between the tension and joint angle. Here, we experimented with various tension conditions to see how the movement of the finger changed under different tension conditions. For loading of each actuation tendon, the maximum tension magnitude that maximizes the finger movement was initially measured. As a next step, the joint angle of the index finger was measured in a condition where the tension of one actuation wire was gradually increased while sustaining the tension of other actuation wires at 0%, 33%, 66%, and 100% of the maximum tension. The second experiment was conducted under free motion, which is a motion without contact with other objects. Finally, using the results of two experiments, various objects were grasped with three major grasps.

## 3. Results

### 3.1. Kinematic System Identification: Estimation of the Relationship between Joint Angle and Fingertip Posture

**Figure 9:**
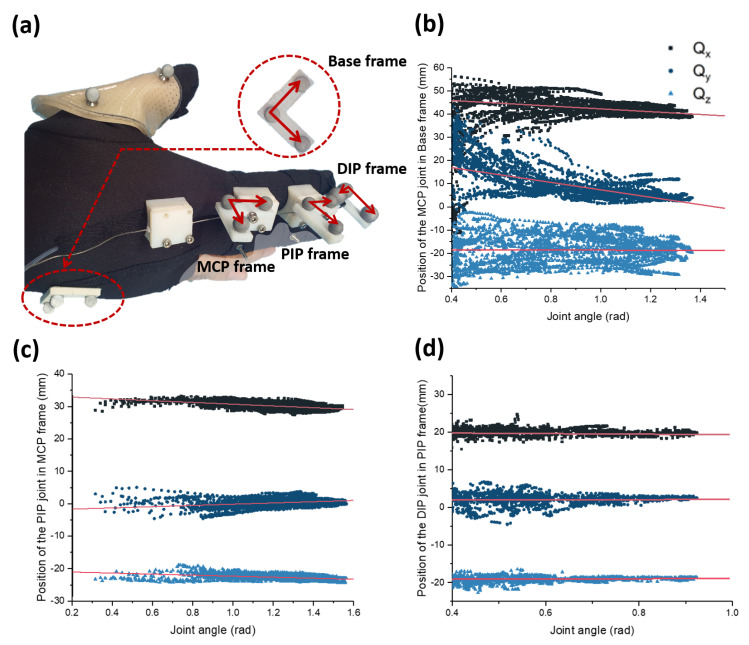
Experimental results about the position of the finger joints found using a vicon motion capture system. (**a**) The marker position used to measure the joint position. With this marker setup, the position of the MCP, PIP, and DIP joint are measured, as shown in (**b**–**d**). Since the joints move as the joint angle changes, the positions of the joints are expressed in terms of the angle of the joints.

The first result examines kinematic system identification, which is designed to find the joint position. The overall experimental setup is depicted in Figure 9a. Here, three markers are attached to each phalange. In addition, three markers are attached to the back of the hand, resulting in a total of 12 markers attached. Using this experimental setup and a vicon motion capture system, each joint position is obtained as shown in Figure 9b–d. Here, the position of the joints is described in terms of joint angle because human joints move when the joint angle changes. This is because human joints are not pin joints; human joints are usually called *rolling contact* joints. In these joints, the bone rotates along the surface, while sustaining the contact with other bones. Here, the position of the joint is expressed with respect to the marker frame, which is attached to the bone in the proximal part of the joint. For example, the position of the DIP joint is expressed with respect to the proximal phalanx.

In order to use the measured data in other analysis, linear regression between the joint rotation and the joint position was performed. Table 1 is the result of the linear regression with the data shown in Figure 9. The parameters in the table indicate the gradient and the y-intercept of X, Y, and Z position of each joint with respect to the rotation angles. For example, X value of MCP joint position can be expressed as −6.05×(joint angle)+48.78. Here, the X, Y, and Z position of MCP, PIP, and DIP joints are expressed in the Base, MCP, and PIP frame respectively (Figure 9a). Since the joint positions have a linear relationship with the rotation angles, we can easily estimate the X, Y, and Z values by using the parameters offered in Table 1. From these joint positions, we can also estimate the length of each phalange, which is the size of the vector pointing from one joint to another, by converting all X, Y, and Z values with respect to the Base frame coordinates.

### 3.2. Stiffness Parameter Estimation—Estimation of the Relationship between Tension and Joint Angle

**Figure 10:**
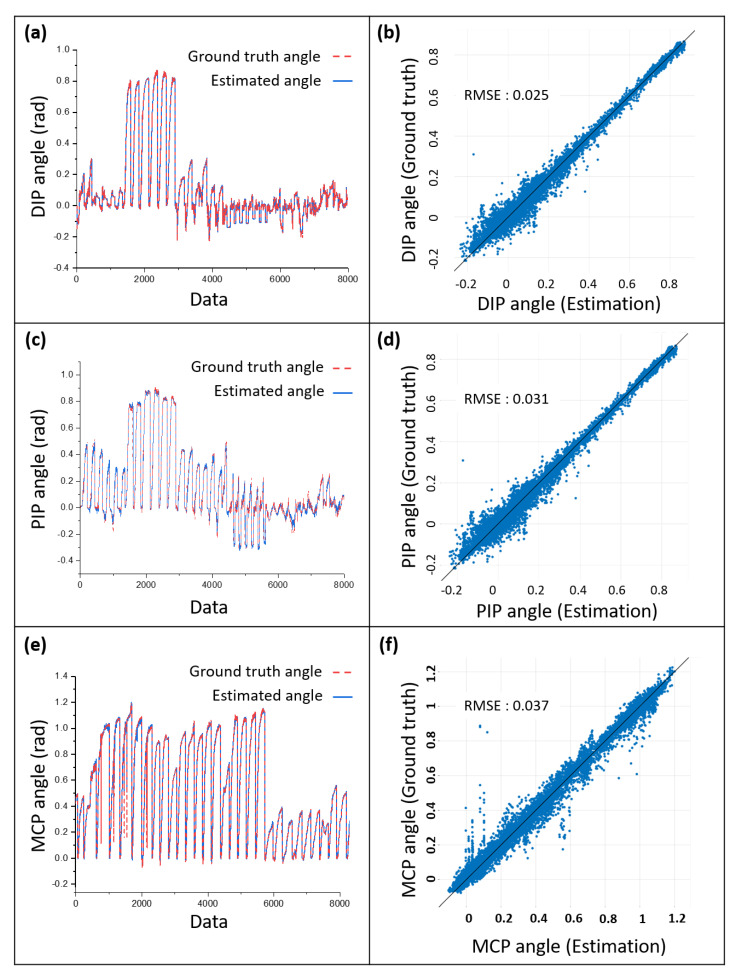
Experimental results of the stiffness parameter estimation that shows a relationship between the joint angle and the wire tension. (**a**,**c**,**e**) are plots of the ground truth angles measured by Vicon cameras and estimated angles using the wire tension and wire stroke. (**b**,**d**,**f**) are comparisons between the estimation and the ground truth. The RMS error indicates the disparity from the y = x relationship.

For the stiffness parameter estimation, we performed experiments to obtain the relationship between wire tension, wire stroke and joint angle. In order to determine the relationship, motor encoder, motion data, and loadcell data was measured simultaneously. The relationship was obtained using Gaussian Process Regression; the results are shown in Figure 10. In this figure, (a), (c) and (e) show the tendency of the joint angle along with the regression results. Here, the x axis of the graphs means the number of data; the number of data in x axis means that i-th row of the x axis is i-th data in the data set. In order to show the accuracy of estimation, the relationship between estimated angle and ground truth angle is compared as shown in Figure 10b,d,f. As the root mean square error (RMSE) in the figures show, the proposed estimation fits well in the ground truth angle.

**Figure 11:**
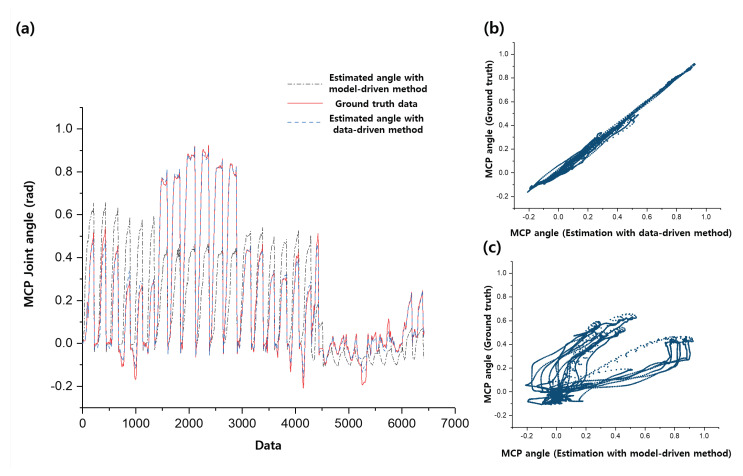
Results of estimation that shows relationship between joint angle and wire tension. (**a**) shows comparison of the ground truth angle with the estimated angle from data-driven method and the estimated angle from model-driven method; (**b**) and (**c**) each show response plot of the data-driven method and model-driven method respectively.

In order to show the effectiveness of the stiffness parameter estimation, we also included additional result of comparison between the proposed estimation and the model-based estimation as shown in Figure 11. As noticed in the Introduction section, since it is difficult to consider the elongation of the robot body or the human joint stiffness in modelling, we used constant value of stiffness and ignored the elongation of the robot body. This model-based estimation is derived using the result of Equation (1) and Equation (21).

### 3.3. Grasp Posture and Range of Motion

**Figure 12:**
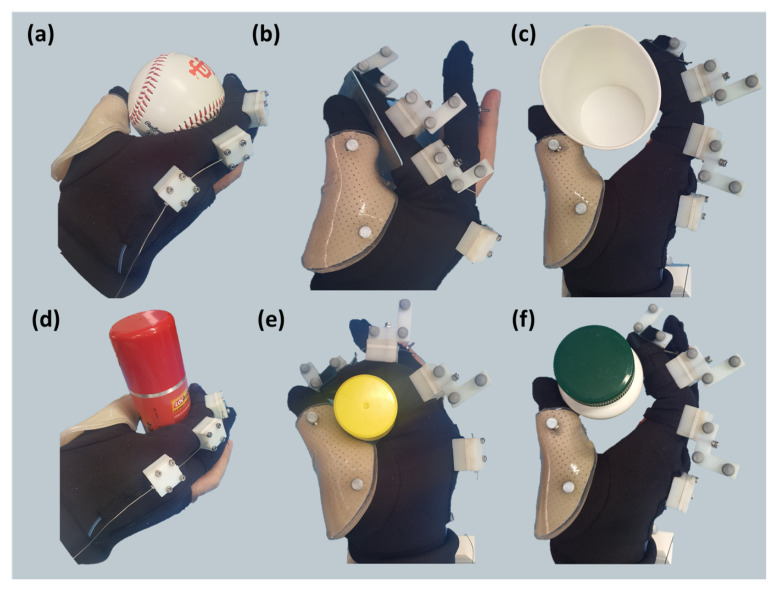
Various postures with the assistance of Exo-Index. With assistance of the proposed robot, three types of grasp postures are established depending on the object size and shape. (**a**) and (**d**) show the posture using the Exo-Index. For more detail, (**b**) shows how card is grasped using narrow pinch while (**e**) shows how glue is grasped with caging. In addition, (**c**) and (**f**) show wide pinch and the grasped objects are paper cup and bottle.

Based on the results from both kinematic and stiffness parameter estimation, several grasp postures can be made using Exo-Index. According to the object shape and size, an appropriate grasp strategy was selected. As mentioned in the introduction, three types of grasp were established, with assistance of Exo-Index. Grasp postures, which are constructed with the proposed robot according to the object shape and size, are shown in Figure 12. As can be seen in the figure, even when supporting only the index finger, it is possible to hold various objects.

**Figure 13:**
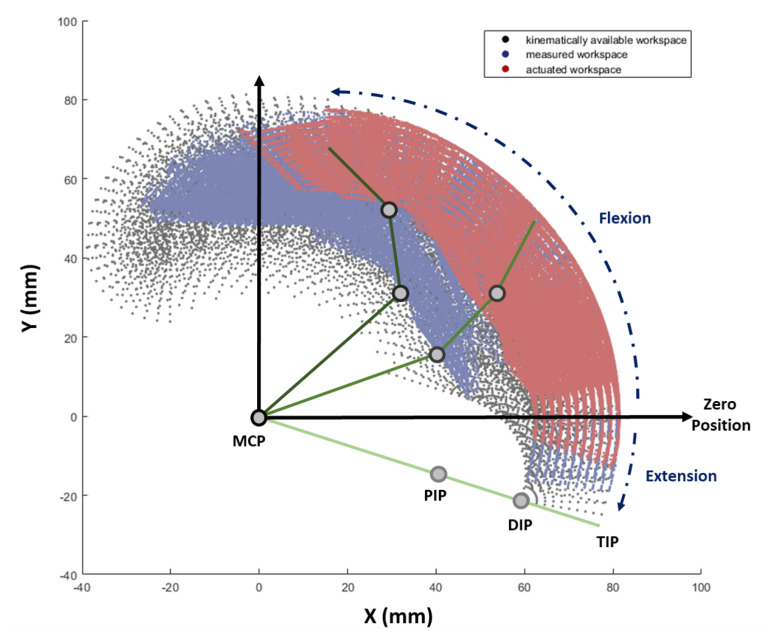
Workspace of the distal phalange under three different motions. Black workspace indicates a kinematically available workspace which is calculated by all possible values in the ROM of the MCP, PIP, and DIP joints. Blue workspace indicates a workspace which is measured by a spontaneous motion of a non-disabled person. The workspace reached by actuating Exo-Index is represented in red. The X and Y axes are coordinates located in the MCP joint and are co-planar with the finger, where X axis is the direction of the proximal phalange when MCP is at zero position and Y axis points at the palmar direction.

For more quantitative results about motion, range of motion (ROM) was measured to determine how much the Exo-Index can assist. ROM generated by spontaneous movement was compared with that generated by robot assistance; the results are expressed in Table 2. Using the obtained ROM, the workspace of distal phalange was obtained, as shown in Figure 13. In this analysis, a control group was set as the kinematically possible workspace, a workspace that is calculated by all values in the ROM range. The spontaneous workspace was obtained using an experimentally measured joint angle. Besides, actuated workspace was measured using assisted motions with Exo-Index.

Since the workspace in Figure 13 is compared using a graphical tool, additional comparison was conducted, as shown in Table 2. Here, each area of the workspace was calculated using a simple Monte Carlo method [34]. As the results show, the workspace when the proposed robot assists is 64.08% of the workspace that is measured when a non-disabled finger is moved spontaneously.

## 4. Discussion

This research proposes a method to identify both kinematic and stiffness parameters of a wearable robot system. In order to verify whether the obtained information is useful in wearable robot study, a novel wearable robot named Exo-Index was designed. As shown in the results, it was possible for the Exo-Index to make three kinds of grasps; the grasp changes depending on the object type. Since the relationship between actuation information, joint angle, and position of the finger is known, it was possible to make a suitable grasp in response to the object status.

The robot proposed in this research has one primary difference from other robots; it assists only a single finger, using three actuators. In contrast, other robots have been developed to assist several fingers in making a grasp posture. Our main reason for concentrating all actuators on a single finger comes from our hypothesis, which is: “For grasping an object using a limited numbers of actuators, controlling the position of a single finger could be a better strategy than controlling a larger number of fingers with coupled motion.” Since the net force applied on the object should be zero for a stable grasp, increasing the number of fingers, without addressing controllability could cause an unwanted situation. Therefore, we fixed the thumb in a specific position and only controlled the index finger.

The attempt to assist using only a single finger is informed by work in previous robotic gripper studies, which have examined the use of two fingers [35,36,37]. Numerous studies proved that using two fingers is sufficient for grasping numerous objects. These two-finger grippers usually make two kinds of grasp modes: parallel grip and caging grip. When the gripper uses a parallel grip, two fingers face each other; therefore, the force closure can be easily achieved. On the other hand, in the case of caging, the gripper structurally prevents the escape of the object by wrapping it with the fingers. These two grips have different aspects—A parallel grip is more accurate because estimation of the force between the object and the gripper is quite easy. However, it is relatively weak to external disturbance due to its limited force direction. On the other hand, the caging grip shows a stable grasp by applying force in various directions; however, in this case, the grasp could be inaccurate because the force applied on the object is difficult to estimate.

Inspired by these prior studies and their grasping strategies, our study also tried to differ the grasp types according to the object shape and size. When the object was small and light, the robot assisted by using a narrow pinch; a wide pinch was used to grasp relatively large and light objects. When the grasp requires an ability to sustain its posture against the external disturbance, caging was used. As shown in the results (Section 3), it was possible to grasp various objects using this proposed grasp strategy. As shown in the results, assisting a single finger can be a sufficient strategy for grasping objects in daily living.

## 5. Conclusions

The main goal of the research is to estimate the hand posture in tendon-driven soft hand wearable robot application using tension sensors and encoders attached to the actuator. The proposed method is derived using data-driven method named Gaussian Process Regression (GPR) and the result of the method shows that it is sufficient to estimate the posture without additional sensors at the wearing part. The RMS error of the estimated joint angle using tension sensor and motor encoder is about 0.03 rad as shown in Section 3.2, which is quite similar to the result of other previous research; The error of the estimation using IMU sensors were reported as 0.027 rad [1].

Besides, the proposed research also shows the method of finding the exact location of human joint with vicon motion capture system. Since the markers used in the vicon system is attached not at the human joint but on the skin of human body, additional process of finding joint axis is executed as shown in the Section 3.1. Using the concept of Product of Exponential (POE), it was possible to find out the accurate position of the joint axis. The result of exact location of human joint was used to derive the exact ground-truth angle, which is required in the process of finding the relationship between joint angle and wire tension.

Although the proposed robot shows sufficient performance in estimation, there were several limitations to this research in a practical issue. First, the proposed robot system cannot configure the object information (e.g., shape, size, and weight) on its own, because the system does not contain any vision sensors. Therefore, it is impossible for this robot to decide the grasp posture depending on the object status. In this initial work, the grasp was decided by the user‘s opinion. Further, when human properties change, the kinematic and stiffness parameter estimation must proceed again; this means that users of Exo-Index have to measure their motion with a vicon motion capture system for setup of the robot.

In order to address the limitations outlined above, our next step in this research will be to include an RGBD camera in the robot system. Using an RGBD camera, it will be possible to identify the status of both the object and the hand. For this method, our goal is to make a learning algorithm that not only distinguishes the object and the hand, but also estimates the object status (e.g., size and shape) and the hand status (e.g., joint angle and joint position).

## Figures and Tables

**Table 1:** Index finger joint position (mm) and finger phalange length (mm). G in the table indicates a gradient of the regression result and the Y means Y-intercept of the regression.

Joint	X			Y			Z			Length
	G	Y	RMSE	G	Y	RMSE	G	Y	RMSE	(mm)
MCP	−6.05	48.78	9.19	−16.04	23.73	5.37	−0.27	−18.13	5.97	42.64
PIP	−2.72	33.58	0.92	1.91	−1.94	1.40	−0.13	−20.64	0.80	20.14
DIP	−0.71	20.41	0.97	0.26	2.05	1.71	0.13	−19.19	0.83	18.72

**Table 2:** Range of motion (ROM) and workspace of each motion. In the table, K.M. means kinematically possible motion, S.M. means spontaneous motion, and A.M. means assisted motion. Since concept of K.M. is only used in workspace analysis, the ROM of K.M. is blank.

	MCP (rad)		PIP (rad)		DIP (rad)		Workspace (mm^2^)
	Max	Min	Max	Min	Max	Min	
K.M.	-	-	-	-	-	-	8712.30
S.M.	1.34	−0.35	1.56	0.08	0.92	0.07	5884.10
A.M.	32.90	21.27	0.88	3.11	4.55	1.48	3770.80

**Figure A1:**
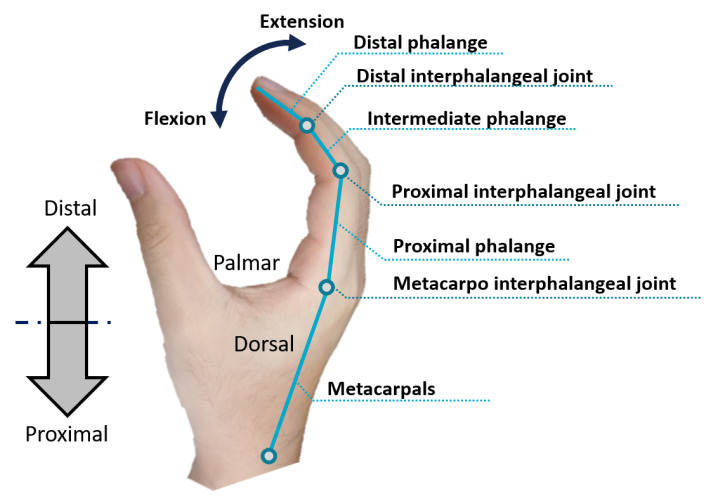
Brief explanation of how the parts of the hand are represented.

## Abbreviations

The following abbreviations are used in this manuscript:

MCP joint	Metacarpophalangeal joint
PIP joint	Proximal interphalangeal joint
DIP joint	Distal interphalangeal joint
PoE	Product of exponential
A.F.	All Flexor
A.E.	All Extensor
M.F.	MCP Flexor
RMSE	Root mean square error
DOF	Degree of freedom
GPR	Gaussian Process Regression

## Appendix A. Anatomic Expressions of the Human Hand

Nomenclature of the joints and phalanges of the hand is described as shown in Figure A1. In the case of the index finger, the distal interphalangeal joint (DIP joint) and the proximal interphalangeal joint (PIP joint) have one degree of freedom (flexion/extension motion), while the metacarpo interphalangeal joint (MCP joint) has two degrees of freedom (flexion/extension and abduction/adduction motion). Also, the word distal is used to describe the direction away from the body, while the word proximal is used to express the opposite direction, which is the direction towards the body. To express the palm side of the hand, the word palmar is used, while the other side of the hand is called the dorsal side.

When a finger moves, the motion is usually expressed in three directions. In the direction of grasping, the motion is usually expressed as flexion, while a motion in the opposite direction is called extension. In addition, if a tendon is used in flexion, it can be roughly called a flexor, while an extensor is used to designate a tendon that is used in extension. Spreading between fingers is called abduction, while the opposite is called adduction. The last motion is called internal rotation; in this motion, the finger itself rotates.
